# Caloric restriction delays early phases of carcinogenesis via effects on the tissue microenvironment

**DOI:** 10.18632/oncotarget.16421

**Published:** 2017-03-21

**Authors:** Erika Cadoni, Fabio Marongiu, Maura Fanti, Monica Serra, Ezio Laconi

**Affiliations:** ^1^ Department of Biomedical Sciences, Unit of Experimental Medicine University of Cagliari-Italy, Cagliari, Italy

**Keywords:** aging, microenvironment, caloric restriction, pre-neoplastic hepatocytes, carcinogenesis

## Abstract

Caloric restriction (CR) is an effective and consistent means to delay aging and the incidence of chronic diseases related to old age, including cancer. However, the precise mechanisms responsible for the beneficial effect of CR on carcinogenic process are yet to be identified.

In the present studies the hypothesis was tested that the CR might delay carcinogenesis via modulatory effects exerted on the age-associated, neoplastic-prone tissue microenvironment. Using a well characterized, orthotopic cell transplantation (Tx) system in the rat, preneoplastic hepatocytes isolated from liver nodules were injected into either old syngeneic rats fed *ad libitum* (AL) or animals of the same age given a CR diet (70% of AL feeding). Analysis of donor-derived cell clusters performed at 10 weeks post-Tx revealed a significant shift towards smaller class sizes in the group receiving CR diet. Clusters comprising more than 50 cells, including large hepatic nodules, were thrice more frequent in AL vs. CR animals. Incidence of spontaneous endogenous nodules was also decreased by CR. Markers of cell senescence were equally expressed in the liver of AL and CR groups. However, higher levels of SIRT1 and FOXO1 proteins were detected in CR-exposed livers, while expression of HDAC1 and C/EBPβ were decreased. These results are interpreted to indicate that CR delays the emergence of age-associated neoplastic disease through effects exerted, at least in part, on the tissue microenvironment. Nutrient-sensing pathways might mediate such modulatory effect.

## INTRODUCTION

Neoplastic disease is inextricably associated with aging. Five out of six cancer-related deaths occur in patients aged 60 years and older [1]. However, the intimate nature of this association is yet to be fully clarified. An important concept emerging from the literature is that aging and cancer do not merely represent two chronologically parallel processes, but they share relevant pathogenetic mechanisms [2–5]. Along these lines, in a recent study we have provided evidence to indicate that aging promotes the growth of pre-neoplastic cells through alterations imposed on the tissue microenvironment, i.e. by generating an age-associated, neoplastic-prone tissue landscape [3]. Similarly, Henry et al. have reported that aging-associated inflammation promotes selection for adaptive oncogenic events in B cell progenitors [2]; it was proposed that cell competition may in fact drive the emergence of oncogenically altered cells in a background of age-induced decline in tissue fitness, in a process that has been referred to as “adaptive oncogenesis” [4, 6].

The notion that age-associated tissue changes may play a direct role in the origin of neoplasia has far-reaching implications. It suggests that strategies aimed at modulating the rate of aging may have a direct impact on early and/or late steps of neoplastic disease, i.e. the quest for a longer lifespan may coincide, at least in part, with the goal to defer the occurrence of cancer [7, 8].

A most effective and consistent means to delay aging is by reducing caloric intake compared to *ad libitum* (AL) feeding. Caloric restriction (CR) is indeed the most studied and reproducible non-genetic intervention known to extend lifespan in organisms ranging from unicellular yeast to mammals [9–10], including non-human primates [11], although the latter observation is disputed [12]. On the other hand, it is also well documented that CR exerts a beneficial effect on the incidence of chronic diseases related to old age, including cancer, consistent with the notion that changes occurring during the aging process may bear direct relevance to the pathogenesis of neoplasia. However, the precise mechanisms responsible for the CR-induced delay on carcinogenic process are yet to be identified.

Based on the above, in the present studies we tested the hypothesis that the modulatory effect of CR on age-associated neoplastic disease might be related, at least in part, to a CR-induced delay in the emergence of age-related tissue alterations promoting the growth of pre-neoplastic cells [3].

Using a well characterized, orthotopic cell transplantation system in the rat, we report that when pre-neoplastic hepatocytes were infused in aged animals exposed to either AL o CR diet, their growth was significantly reduced in the latter group.

## RESULTS

### The growth of transplanted normal hepatocytes in the liver of rats exposed to long-term CR

**Figure 1:**
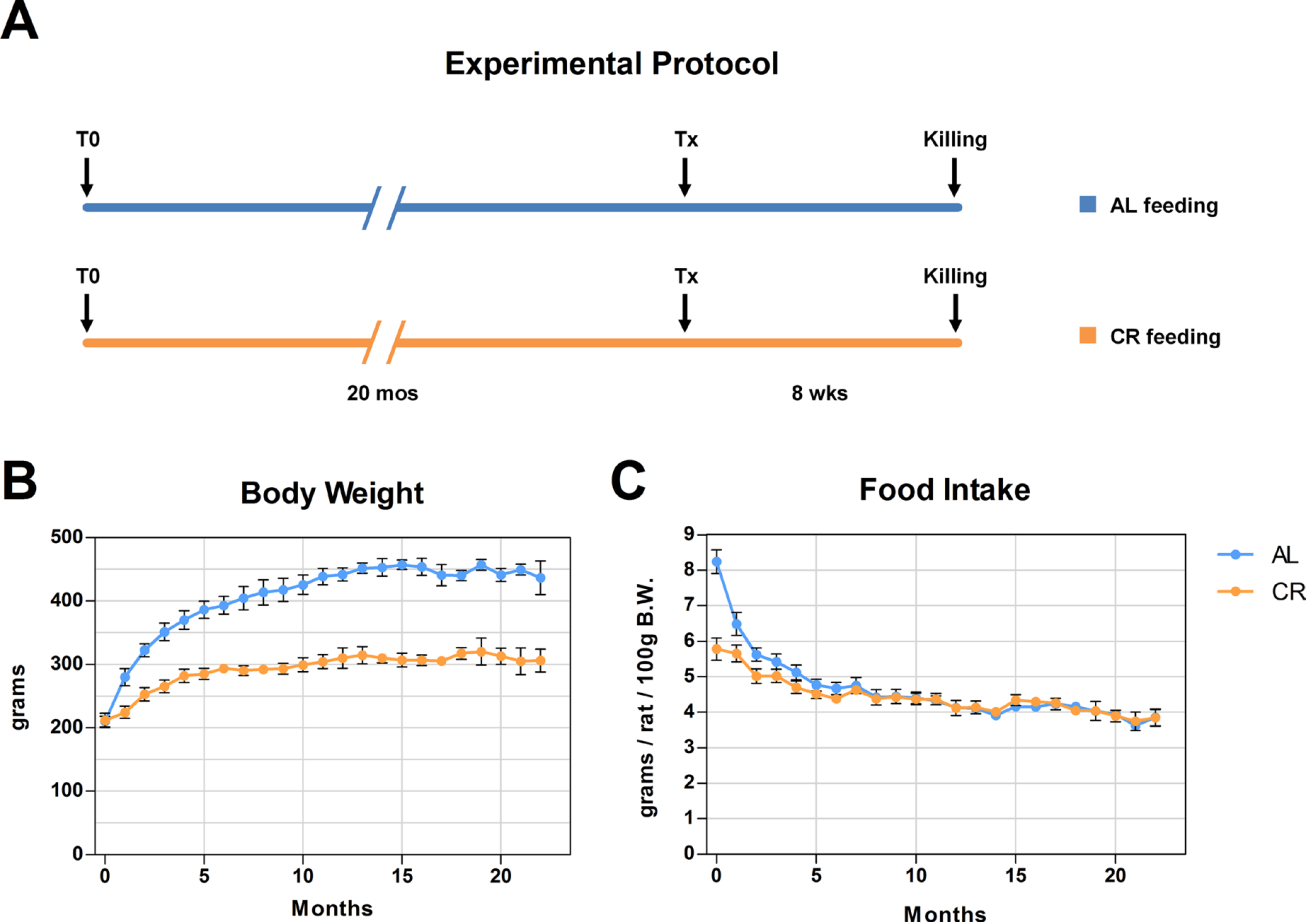
Experimental protocol is presented in (**A**) Animals were fed either AL or CR diet and were the transplanted with normal hepatocytes. Growth curves and food intake in both groups are reported in panels (**B** and **C**).

**Figure 2:**
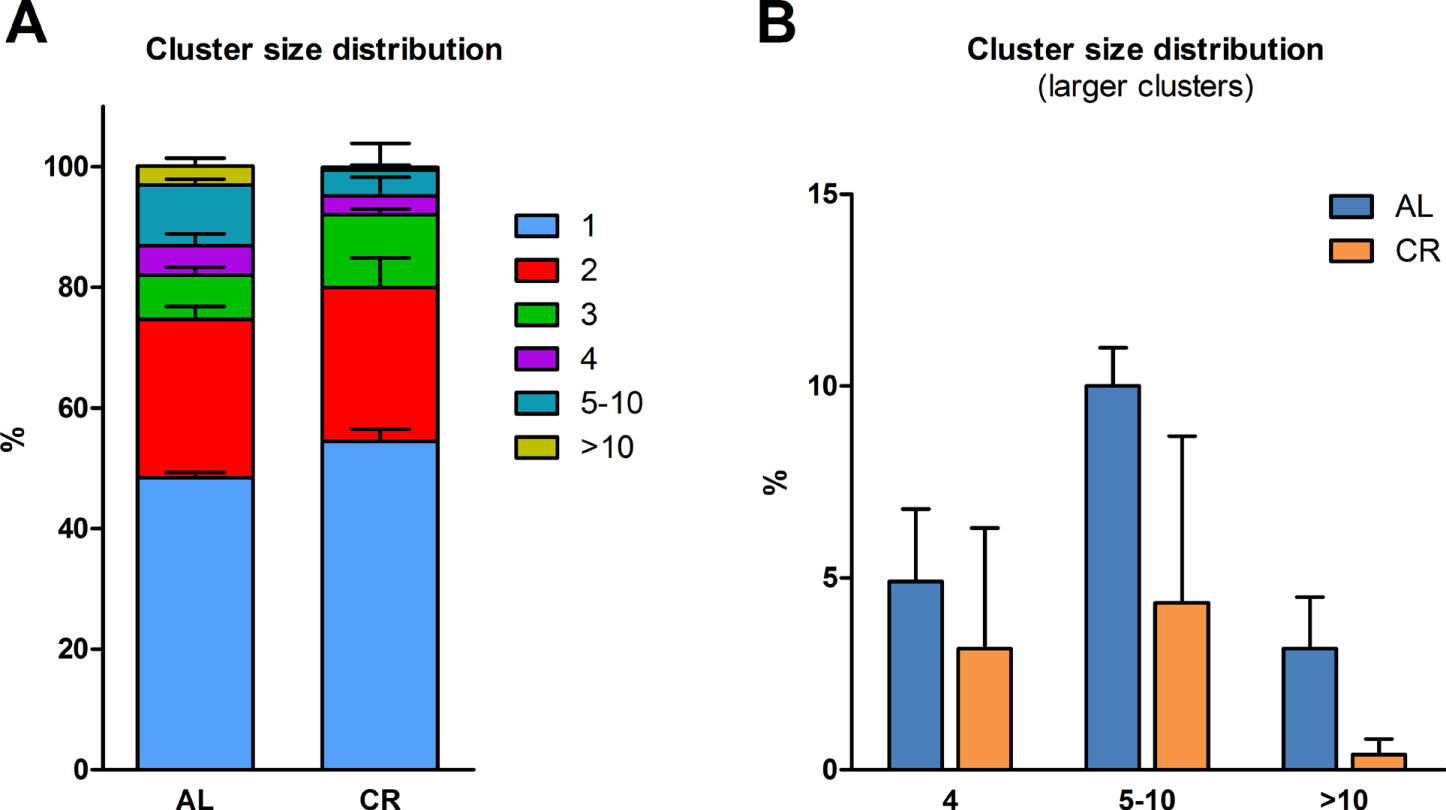
Size distribution of donor-derived normal hepatocyte clusters following transplantation of isolated cells in animals given AL or CR diet (**A**) clusters of any size; (**B**) clusters comprising 4 cells or larger. Each cluster was analyzed three-dimensionally (see Experimental procedures for details)

In a first study, we explored the fate of normal hepatocytes transplanted into the liver of aged animals exposed to either AL or CR. This was largely based on our earlier observation indicating that the tissue microenvironment of the aged rat liver supports the clonal expansion of hepatocytes isolated from a normal young donor, while the liver of young recipients was not permissive [13]. Thus, we tested whether CR exerted any modulatory effect on the clonogenic potential of the aged liver milieu on transplanted normal hepatocytes. To this end, the dipeptidyl-peptidase type IV-deficient (DPPIV^−^) rat model for cell transplantation was used. In this system, the recipient animals lack DPPIV enzyme activity, while donor cells are taken from a DPPIV-expressing, syngeneic animal, allowing for the rapid detection of the injected cell progeny in the host tissue through a simple histochemical staining [13]. Two-month old Fischer 344 rats of the DPPIV^−^ strain were fed either AL or CR diet for 20 months and were then transplanted with normal hepatocytes. Both groups were killed 8 weeks later (see experimental protocol in Figure 1A). Body weights during the experiment in AL and CR-treated animals are presented in Figure 1; final body weights were 304 ± 17 vs. 433 ± 27 g, respectively, in line with data reported in the literature [14]. Absolute food intake was reduced to 70% of AL level in CR group, according to protocol. However, the relative amount of food consumption (expressed per body weight) became comparable in the two groups after about 3 months of treatment and until the end of the experiment (Figure 1C). The size distribution of donor-derived hepatocyte clusters obtained from 3D analysis of the liver sections in animals receiving AL or CR diet is reported in Figure 2. Single cells and doublets were by far the most represented size classes, as expected, with no intergroup differences. On the other hand, clusters comprising 5 to 10 DPPIV^+^ hepatocytes were twice more frequent (10.0 vs 4.4%) in the former group and clones larger than 10 cells were 8 times more common in AL vs. CR group (Figure 2B), although differences did not reach statistical significance.

### The growth of transplanted nodular hepatocytes in the liver of rats exposed to long-term CR

**Figure 3:**
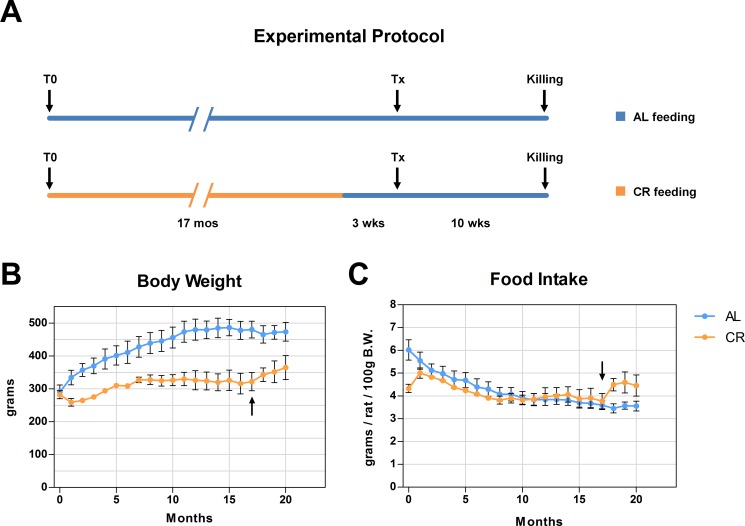
(**A**) reports the detailed experimental protocol. Animals were fed either AL or CR diet for 17 months. Both groups were then continued with AL feeding and three weeks thereafter they were the transplanted (Tx) with pre-neoplastic hepatocytes. All animals were killed 10 weeks after Tx. Growth curves and food intake in both groups are reported in panels (**B** and **C**) arrows in panels B and C indicate the shift of CR group to AL feeding.

**Figure 4:**
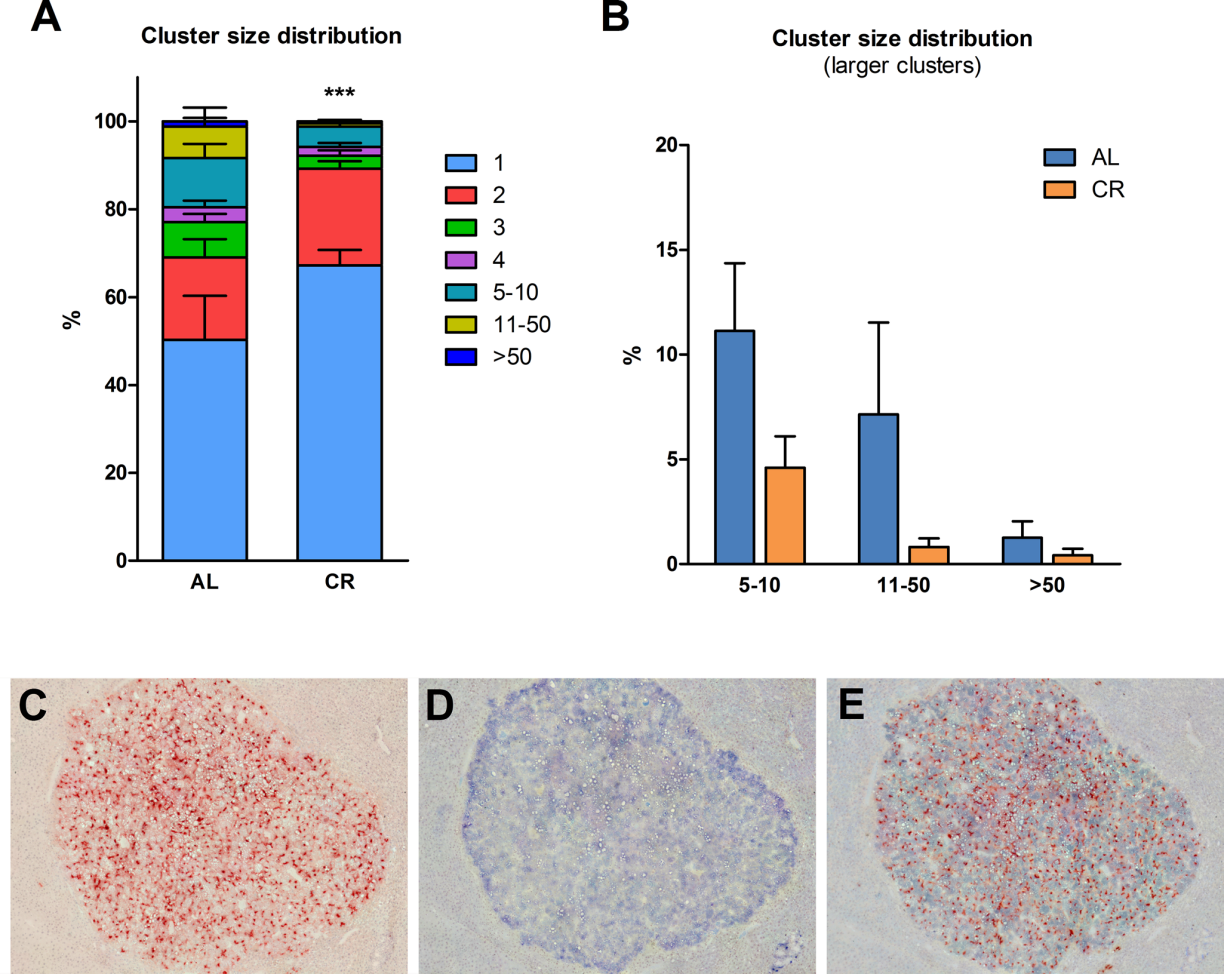
Size distribution of donor-derived nodular hepatocyte clusters following transplantation of isolated cells in animals given AL or CR diet (**A**) clusters of any size; (**B**) clusters comprising five cells or larger. Each cluster was analyzed three-dimensionally (see Experimental procedures for details). Significantly different from control, ***p < 0.001. (**C, D** and **E**) serial section of a donor-derived hepatocyte nodule in the liver of AL fed animal. (C and D) show histochemical staining for DPPIV and immuno-histochemical staining for GST-P, respectively; in (E) staining for the two markers was combined. Original magnification: 40x.

As previously mentioned, recent results from our laboratory have indicated that the aging process fuels carcinogenesis via alterations imposed on the tissue microenvironment [3]. In light of this observations and based on results presented in the preceding paragraphs, we then tested the possibility that the delaying effect of CR on carcinogenesis might be exerted, at least in part, via modulation of the neoplastic-prone microenvironment associated with aging. To this end, nodular (pre-neoplastic) hepatocytes isolated from a DPPIV^+^ donor were transplanted into old rats lacking DPPIV and fed either AL or a 70% CR diet for 17 months (see experimental protocol, Figure 3). In order to avoid possible direct effects of the different dietary regimens on transplanted cells, both groups were allowed AL feeding starting 3 weeks prior to transplantation and until the end of the experiment. Results are presented in Figures 3 and 4. Growth curves were similar to those obtained in the former study, except for the last 4-month segment when both groups were fed AL diet (Figure 3). Final body weights and relative liver weights were significantly lower in CR-fed animals (364 ± 36 vs. 473 ± 28 and 2.98 ± 0.08 vs. 3.48 ± 0.11%, respectively), despite their exposure to AL feeding during the last 13 weeks of the study (Figure 3A). Figure 4 reports the size distribution of donor-derived clusters in AL and CR fed rats killed 10 weeks post-transplantation. Cluster cell number was measured three-dimensionally through the analysis of serial sections. Clusters comprising more than 5 cells were more frequent in AL group. More specifically, clusters ranging from 5 to 10 cells represented 11.1% of total clusters in AL vs. 4.6% in CR animals. Clusters of 10 to 50 cells in size were 7.7% in AL and less than 1% in CR group. Finally, rare clusters comprising more than 50 cells, including large hepatic nodules (Figure 4C–4E), were thrice more frequent in AL vs. CR animals.

### CR decreases the size of “spontaneous” endogenous pre-neoplastic lesions

**Figure 5:**
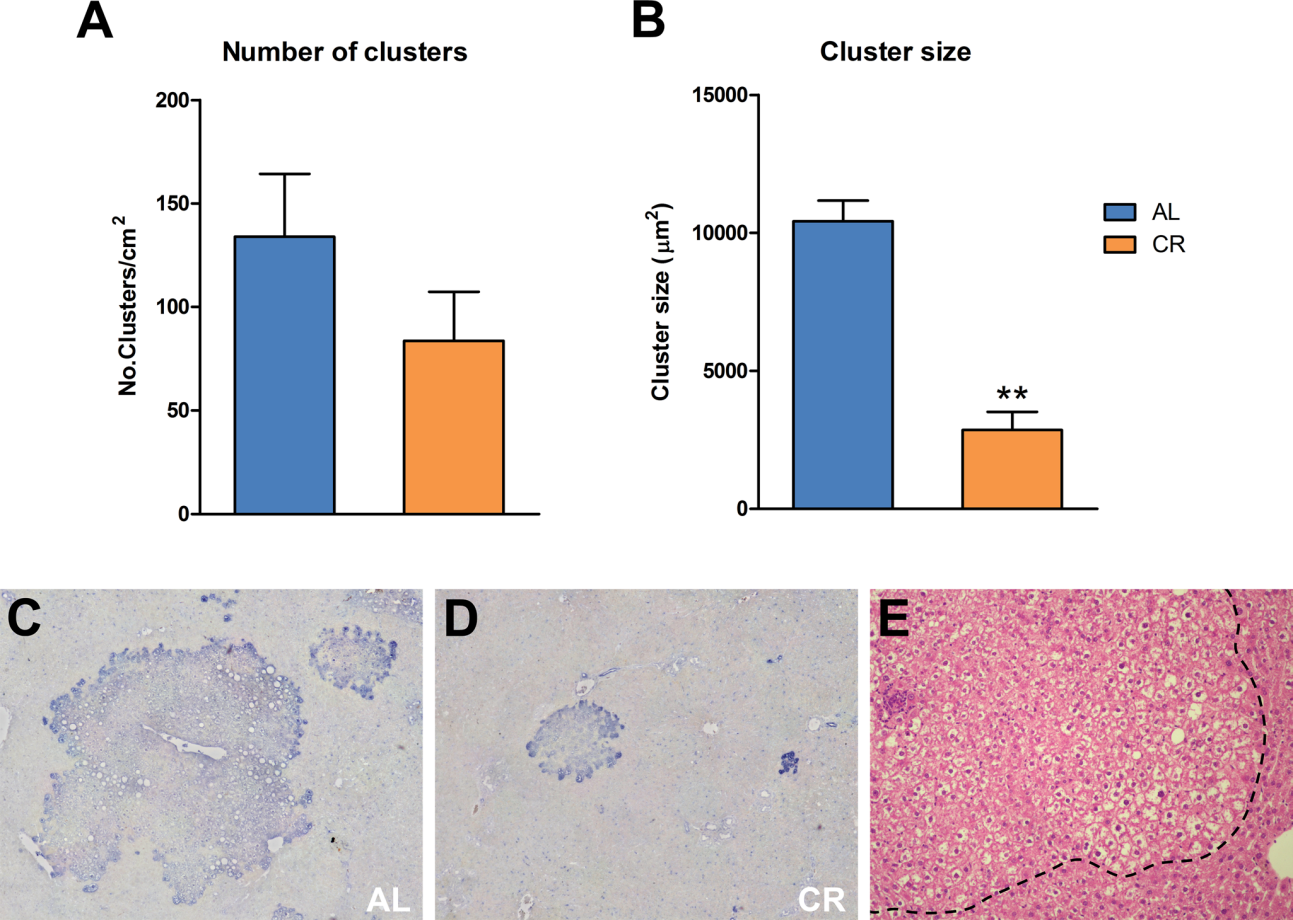
Incidence of spontaneous GST-P-positive foci and nodules in animals fed either AL or CR diet for 17 months, followed by AL feeding for 3 months (see Figure 3 for experimental details) While the number of lesions (**A**) was not significantly different between the two groups, size of GST-P-expressing cell clusters (**B**) was greatly reduced in CR group, as expected. Significantly different from control, **p < 0.01. (**C** and **D**) show immuno-histochemical staining for GST-P in AL and CR liver, respectively, while a typical “ground glass”-appearing focal lesion (outlined by the dotted line) is presented (**E**) Original magnification: 40x.

The beneficial effect of CR on carcinogenesis was also confirmed by analyzing the size of “spontaneous” endogenous glutathione-S-transferase-placental form (GST-P)-positive lesions, which is a common finding in the Fischer 344 strain of rats [14]. While all animals in both groups displayed GSTP-positive foci, the size of these lesions (which were negative for the DPPIV donor marker) was significantly lower in animals given the CR diet (Figure 5A–5E). These findings are in line with previous reports showing that long-term CR delays the development of spontaneous cancers in several models, from rodents to monkeys [9–12, 15, 16] and in several tissues, including liver [17].

### Markers of cell senescence in the liver of rats exposed to long-term CR

Cell senescence has been included among the “hallmarks of aging” [18], although its specific role in the aging process, if any, is yet to be defined. It is now well established that senescence may exert opposing roles in carcinogenesis. In fact, it has been regarded as a fail-safe mechanism to limit the risk of neoplastic transformation following genotoxic insult. At the same time, cellular senescence has been shown to fuel neoplastic process, possibly via the senescence-associated secretory phenotype (SASP), which includes growth factors, pro-inflammatory cytokines and matrix-remodeling enzymes [19–21]. Since CR is known to delay the aging process, we determined whether long-term CR has any effect on the emergence of cellular senescence in liver, given the potential role of this phenotype in the context of the tissue microenvironment. Analysis was performed on samples obtained from experiment outlined in Figure 3.

#### a. Expression of senescence-associated-β-galactosidase

**Figure 6:**
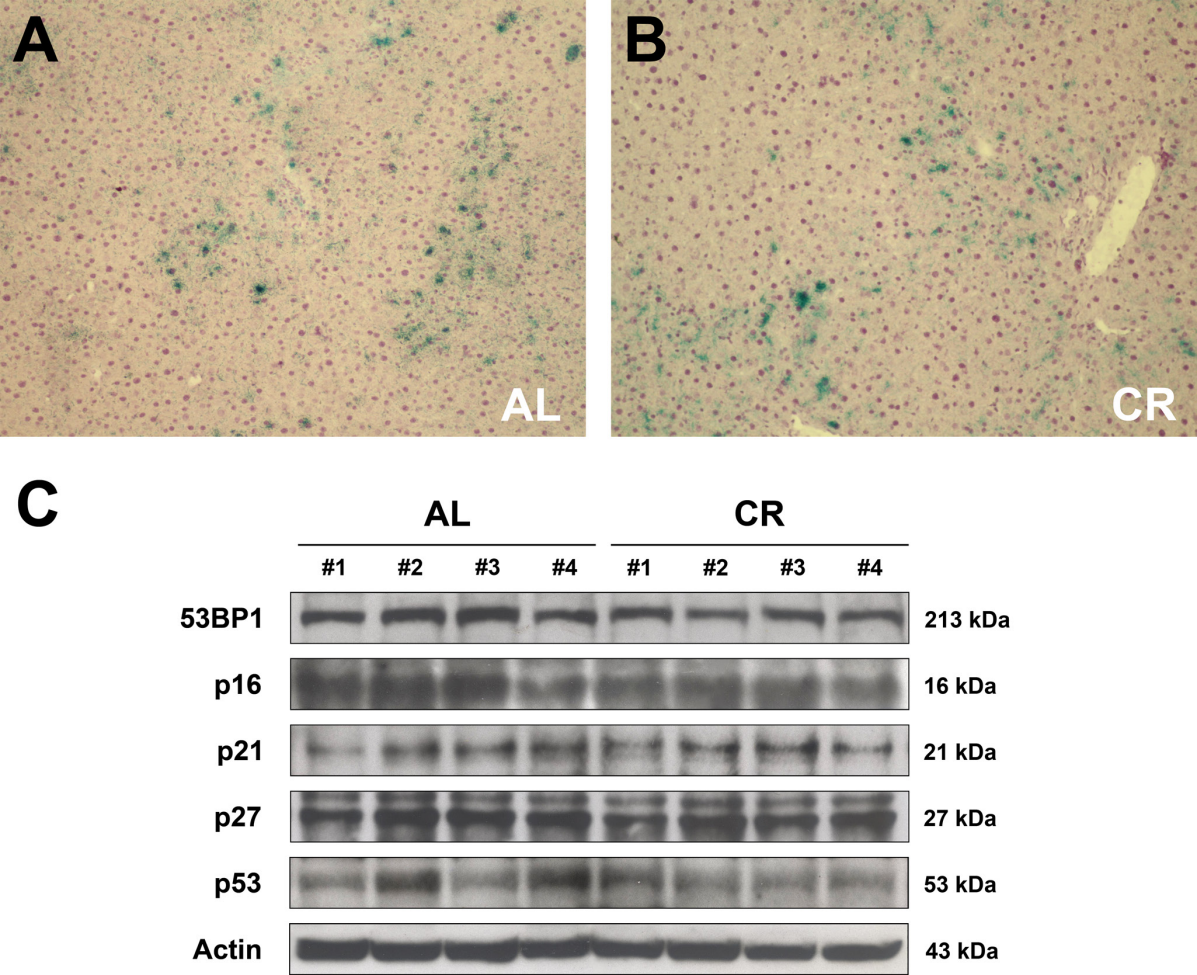
Markers of cell senescence in the liver of animals fed either AL or CR diet for 17 months, followed by AL feeding for 3 months (see Figure 3 for experimental details) (**A** and **B**) report histochemical staining for SA-β-gal in AL and CR groups, respectively. Expression of 53BP1, p16, p21, p27 and p53 is presented in (**C**). No intergroup differences were seen in any of these proteins.

Senescence-associated-β-galactosidase (SA-β-gal) is among the most commonly used markers of cell senescence [22]. Histochemical staining revealed the expression of SA-beta-gal in both groups, fed either AL or long-term CR, with no intergroup differences. This finding was obtained in animals transplanted with normal hepatocytes and was confirmed in groups injected with cells isolated from hepatic nodules (Figure 6A and 6B).

#### b. Expression of markers of DNA damage response

DNA damage is a possible trigger of cellular senescence [23]. Replication errors, telomeres shortening, genotoxic stress, ionizing radiation induce the DNA damage response (DDR), recruiting on the site of the damage the ataxia-teleangiectasia-mutated (ATM) protein, which causes the phosphorylation of H2A histone family, member X (γ-H2AX); this in turn facilitates the assembly of checkpoints and DNA repair factors such as p53 binding protein 1 (53BP1) [24, 25]. Based on these premises, the expression of 53BP1 was analyzed in the liver of rats exposed to either AL or early-onset, long-term CR feeding (see Figure 3 for details). However, no significant differences were observed in the expression of 53BP1 in the aged liver of AL- or CR-fed rats after 22 months of feeding (Figure 6C). Similarly, no evidence of γ-H2AX-positive foci was found in the liver of either groups (data not reported).

#### c. Expression of cell-cycle check-point regulators

Cell senescence is often associated with increased expression of cell-cycle inhibitors such as p16, p21, p27 and p53, which are implicated in check-point regulation. Most frequently, senescent cells over-express p21 and p16, which are components of the tumor-suppressor pathway governed by p53 and RB [26, 27]. Previous studies have also indicated that expression of p21 and/or p27 is increased in the aged liver of rodents [28, 29]. When liver samples obtained from both AL and long-term CR groups were analyzed, no significant differences could be detected in the expression of any of these putative markers of cell senescence. Thus, WB analysis of liver samples showed no differences between AL and CR groups in the expression levels of p21, p27, p16 or p53 (Figure 6C).

### Regulation of nutrient-sensing pathways in the liver of rats exposed to long-term CR

Nutrient-sensing pathways have been established as important mediators of the beneficial impact of CR on aging [30]. Given their central role in tissue metabolism, they could be involved in the observed modulatory effect of CR on the emergence of the neoplastic-prone microenvironment associated with aging. Accordingly, we monitored the expression of key components of nutrient sensing pathways in the liver of rats exposed to either AL or long-term CR feeding.

**Figure 7:**
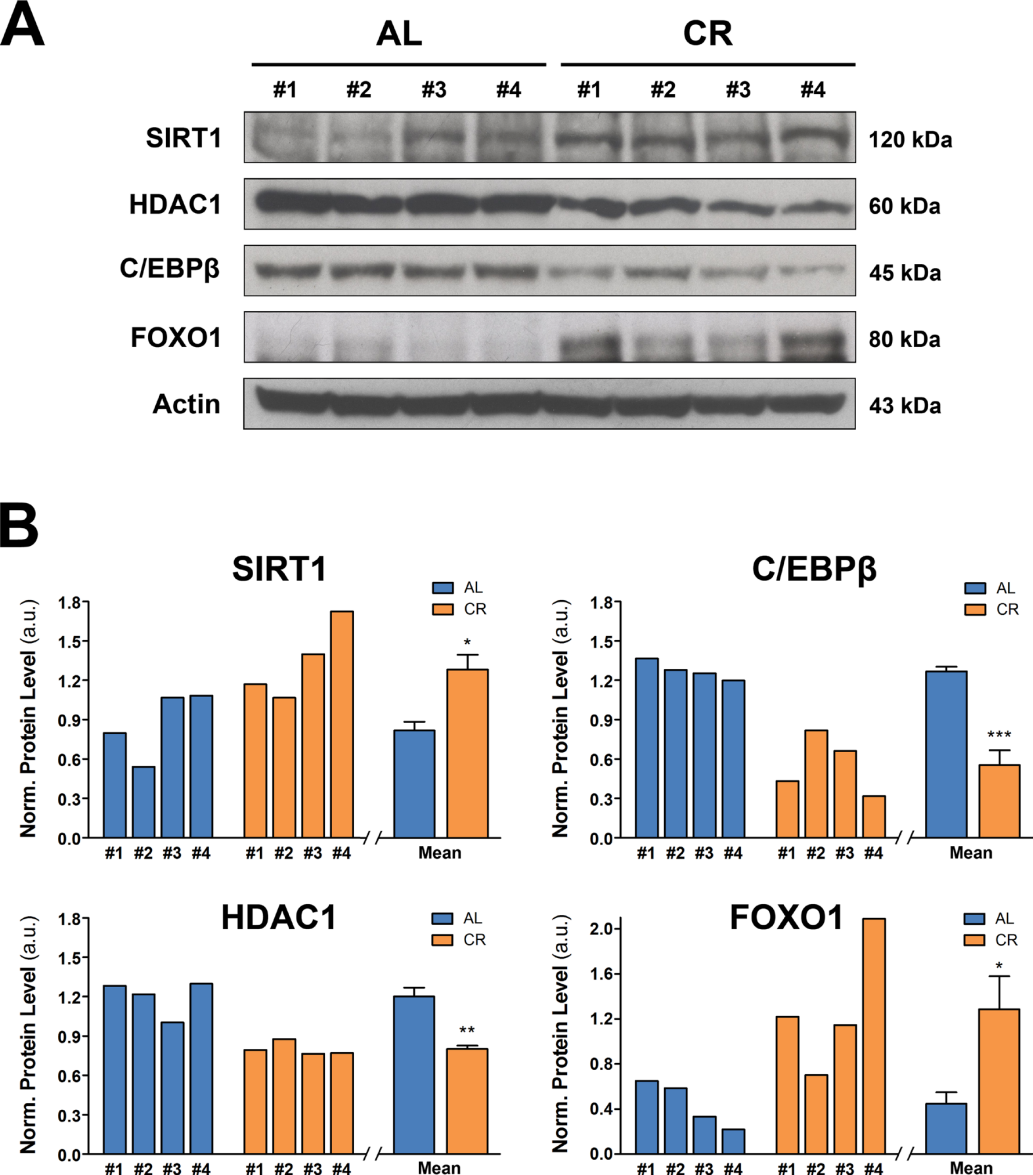
Nutrient-sensing pathways in the liver of animals fed either AL or CR diet for 17 months, followed by AL feeding for 3 months (see Figure 3 for experimental details) (**A**) reports WB analysis for the expression of SIRT1, HDCA1, C/EBPβ and FOXO1, while in (**B**) the corresponding densitometries with reference to actin is presented. Significantly different from control: *p < 0.05; **p < 0.01; ***p < 0.001.

The NAD^+^-dependent protein deacetylase SIRT1 has been implicated as downstream regulator of CR effects in several experimental models and in humans [31, 32]. SIRT1 senses the nutritional state of the cell since its deacetylase activity depends on NAD^+^ availability. It has been shown to decrease in old tissues, including liver, and this change has been linked to the age-associated decline in the regenerative capacity of this organ [33, 34]. Since SIRT1 localizes mainly in the nucleus, we performed WB analysis on nuclear proteins. As shown in Figure 7, it was found that long-term CR increased significantly SIRT1 levels in liver, compared to the very low levels detected in AL fed animals.

SIRT1 decrease in aged liver seems to be mediated by a complex formed by CCAAT/Enhancer binding protein-β (C-EBPβ) and histone deacetylase 1 (HDCA1), which blocks the SIRT1 promoter, thereby preventing its activity [33]. We therefore tested if long-term CR may affect the expression of HDCA1 and C/EBPβ, which have already been shown to be up-regulated in old liver [33]. Analysis of nuclear proteins revealed lower levels of both HDAC1 and C/EBPβ in livers of animals exposed to CR diet compared to AL fed group (Figure 7A and 7B). The pathway of SIRT1 is also involved in the regulation of forkhead box, sub-group O (FOXO) proteins, which have been implicated in the life-extending effect of CR [35]. Indeed, SIRT1 deacetylates FOXOs in response to oxidative stress, protecting cells from oxidative damage [36]. Furthermore, it has been reported that one member of this protein family, FOXO1, mediates, at least in part, the anti-neoplastic effect of CR [37]. Based on this evidence, we determined whether long-term CR affected FOXO1 expression in rat liver, as part of its modulatory effect on the aged tissue microenvironment. Results on nuclear protein analysis are also reported in panels A and B of Figure 7. A significant increase in FOXO1 expression was seen in rat liver from CR-fed animals compared to AL controls, consistent with the increased levels of SIRT1 reported above.

## DISCUSSION

Caloric restriction (CR), the non-genetic intervention known to delay aging in several species from yeast to monkeys, has been shown to retard both spontaneous and chemically-induced neoplastic disease in experimental animals [9–12, 15–17, 38, 39]. Several mechanism have been proposed in the literature to explain the delaying effect of CR on cancer development [8–12]. In the present studies, we report evidence to suggest that long term CR exerts a modulatory effect on the emergence of the neoplastic-prone tissue microenvironment associated with aging, thereby decreasing the risk of cancer. To our knowledge this is the first study focusing on the effect of long-term CR on the tissue microenvironment in relation to neoplastic disease.

We have initially performed a study involving transplantation of normal hepatocytes in rats of 22 months of age that were fed either AL or a CR diet for 20 months. Results gave a clear indication that clusters of donor-derived hepatocytes were larger in AL-fed animals compared to those exposed to CR, suggesting that CR helps to preserve the young phenotypic features of the liver tissue microenvironment. Such interpretation stems from our previous findings indicating that the growth of normal hepatocytes orthotopically transplanted in both young and old recipients is more prominent in the aged liver [13].

We then directly addressed the possibility that CR may impact the evolution of the neoplastic process via effects exerted on the tissue microenvironment. This hypothesis was also grounded on our recent report describing the promoting capability of an aged tissue-microenvironment on the growth of pre-neoplastic hepatocytes [3]. Two groups of rats were fed either AL or a CR diet for 17 months, starting at 8 weeks of age and they were then transplanted with pre-neoplastic hepatocytes. As detailed in the preceding section, results were clear-cut. Larger nodular cell clusters were significantly more common in AL compared to CR fed group, i.e. feeding a CR diet for 18 months was able to reduce the tumor promoting potential of the aged liver microenvironment. Of note, exposure to CR was halted 3 weeks prior to transplantation of nodular hepatocytes, ruling out the possibility that the dietary regimen exerted its effect directly on the pre-neoplastic cell population.

Pertinent to the latter consideration, we also observed a decreased incidence of endogenous hepatic nodules in animals fed the CR diet. The Fischer 344 rat strain is known to be prone to develop various types of “spontaneous” neoplasms, including liver lesions, with advancing age [14]. Interestingly, the decrease in size of endogenous liver nodules in the CR group was of a similar order of magnitude to that observed for donor-derived nodular hepatocyte clusters. This suggests that the delaying effect exerted by CR on “spontaneous” neoplastic disease may in fact be largely mediated by alterations imposed on the tissue microenvironment, i.e. through a modulatory effect on the emergence of the age-associated, neoplastic-prone tissue landscape.

The biological determinants contributing to the increased proneness of the aged tissue environment to carcinogenesis are yet to be defined. It is noteworthy that both normal and pre-neoplastic hepatocytes appear to be more susceptible to clonal expansion when injected into the liver of old animals compared to that of young counterparts [3, 13] or to aged animals exposed to CR, as shown in the present studies. This suggests that similar biological forces drive the growth of both cell types, raising the possibility that a common denominator might be at play.

If this is the case, one might address the issue by exploring the mechanisms sustaining the growth of normal hepatocytes transplanted in the aged host liver [13]. It is well established that the regenerative capacity of the liver declines with age [40]. Moreover, work for our research group has indicated that a cell-autonomous decrease in proliferative proficiency is in fact present in hepatocytes isolated from aged animals [41]. It is therefore conceivable that a decreased proliferative competiveness of the aged hepatocyte may provide a selective advantage for transplanted cells, favouring their expansion, while the same cells do not emerge following infusion into the liver of young animals [3, 13]. Interestingly, it has also been reported that CR (60% of AL diet for 14 months) was able to improve the regenerative response of old Fischer 344 rat liver to partial surgical hepatectomy [42], suggesting that CR may recover, at least in part, the proliferative competiveness of the aged hepatocytes. Thus, the liver microenvironment of the CR-exposed aged rat appears to maintain a higher functional competiveness, which may translate in a less permissive microenvironment for the clonal emergence of either normal or putative altered/pre-neoplastic cells.

We also examined the possible contribution of cell senescence to the delaying effect of CR on carcinogenesis. Senescent cells are considered a hallmark of aging since they accumulate in many tissues of old vertebrate organisms [26, 27]. Although senescence is thought to limit the risk of neoplastic transformation following genotoxic insult, there is now abundant evidence to indicate that it can also fuel carcinogenesis, possibly via SASP components [26, 27]. In light of this possibility, we tested if long-term CR has any effect on the incidence of cell senescence in liver. Livers from AL and CR animals were screened for a range of senescence markers, such as expression of SA-beta-gal, DNA damage response or cell-cycle inhibitors; however, no significant differences in any of these parameters were found between the two groups. Our results are at variance with previous findings in mouse heart, liver, small intestine and kidney, where both short-term and long-term CR were shown to decrease the expression of some senescence markers such as SA-beta-gal, H2AX, p16 and lipofuscin [25]. The reasons for these discrepancies are difficult to evaluate at this point; however, species differences (mouse vs. rats) might be involved. For example, studies in rhesus monkeys, where CR was implemented for 9–12 years, showed no reduction in the expression of senescence markers [43].

At the biochemical and metabolic level, we confirmed and extended several findings related to the effect CR. Thus, levels of SIRT1, which were shown to increase during CR in several tissues in mice [31, 32], were higher in the liver of CR rats compared to AL fed controls. FOXO1, a downstream effector of SIRT1, was also significantly increased in livers of rats exposed to CR, in agreement with results obtained in mice [37]. SIRT1 is downregulated in the aged liver and it has been suggested as a key protein linking aging and liver dysfunction. [33]. On the other hand, HDAC1 is involved in SIRT1 regulation, via formation of a multiprotein complex with C/EBPβ, whose levels have been found increase in old liver as well [44]. Indeed, HDAC1-C/EBPβ complex represses SIRT1 promoter, and this effect has been linked to in the impaired regenerative capacity of the aged liver [33]. In our studies, we observed a decrease in both HDAC1 and C/EBPβ expression in CR livers, indicating that long-term CR improves the functional proficiency of old liver. As referred to above, evidence has indicated that CR ameliorates the proliferative response of the liver to partial hepatectomy [42]. Thus, metabolic changes associated with long-term exposure to CR are consistent with the hypothesis that CR diet appears to delay the emergence of decreased tissue fitness associated with aging, thereby reducing the competitive advantage of transplanted normal and/or pre-neoplastic cells in the liver microenvironment.

In summary, our findings add to current understanding of the pathogenetic mechanisms linking aging, neoplastic disease and the modulatory effect CR on both biological processes. They provide clear evidence to indicate that the delaying effect of CR on carcinogenesis is mediated, at least in part, through CR-induced changes in the tissue microenvironment. More specifically, CR appears to result in the persistence of “young” phenotypic features in the liver tissue, both at biological and at biochemical/molecular level. The overall effect translates in a delayed emergence of the age-associated, neoplastic-prone tissue landscape. Our findings support the notion that strategies aimed at delaying biological aging, such as CR, are also effective in decreasing the risk of neoplastic disease [7, 8, 45].

## MATERIALS AND METHODS

### Animals and food

All animals were maintained on daily cycles of alternating 12 h light/darkness with water available ad libitum. Rats were housed in cages with two rats each, they were fed Purina Rodent Lab Chow diet (Mucedola, Italy) throughout the experiments and received humane care according to the criteria outlined in the National Institutes of Health Publication 86–23, revised 1985. Animal studies were reviewed and approved by the Institutional Animal Care and Use Committee of the University of Cagliari. In order to distinguish donor-derived from recipient cells in the liver, the dipeptidyl-peptidase type IV-deficient (DPPIV-) rat model was used [46]. A colony of DPPIV- F344 rats has been established in our laboratory, at the Department of Biomedical Sciences, University of Cagliari. DPPIV- animals were used as recipients, while donor rats were syngeneic F344, DPPIV+ and were purchased from Charles River, Milan, Italy.

### Dietary regimen

In a first series of experiments, 8-week-old male F344 rats were randomly divided into two groups: a control group receiving food *ad libitum* (AL) and a caloric restriction group (CR) receiving 70% of food consumed by AL-fed rats (Figure 1A). Both groups received the same standard rodent laboratory chow. After 20 months under AL or CR diet, animals were transplanted with normal hepatocytes and killed 8 weeks later.

In a second series of experiments, 8-week-old male F344 rats were similarly divided into two groups, and were given either AL or CR diet (Figure 3A). After 17 months, animals in the CR group were transferred to AL diet until the end of the experiment. Three weeks after the dietary shift, all animals were transplanted with nodular (pre-neoplastic) hepatocytes and killed 10 weeks afterwards. Body weight and food consumption were monitored weekly. The 70% amount of CR diet was calculated weekly based on the average of food consumption in AL rats over the preceding week. The level of 30% restriction in calorie intake was chosen in order to achieve a beneficial effect without causing malnutrition, and was based on numerous reports in literature [47]. Food to CR group was delivered at 1:00 AM through a computer assisted food dispenser. At sacrifice, livers were excised and samples from each lobe were snap frozen (for cryostat sections and protein analysis) or fixed in 10% buffered formalin and embedded in paraffin (for histology and immunohistochemistry).

### Isolation and transplantation of normal and nodular hepatocytes

Normal hepatocytes were isolated from a liver of a male F344 rat expressing DPPIV enzyme activity, according to a standard 2-step collagenase perfusion technique [48, 49]. Hepatocyte nodules were induced according to a well characterized experimental model in the rat [3]. Briefly, two-month old male Fischer 344 DPPIV+ rats were injected with a single dose of diethylnitrosamine (DENA, 200 mg/kg. b.w., i.p., Sigma-Aldrich, St. Louis, MO) followed 2 weeks later, by exposure to a modified version of the Solt and Faber protocol [3], to stimulate the growth of hepatocyte foci and nodules. Such protocol consisted of three consecutive daily doses of 2-acetylaminofluorene (20 mg/kg b.w., given by gavage tube, from Sigma-Aldrich) followed, on the fourth day, by a single administration of CCl_4_ (0.2 ml/kg b.w., by gavage, mixed in corn oil, 1:1 v:v). Six months after the initial treatment livers were perfused as above. Typically, 3 to 5 large (5–10 mm in size) persistent nodules are present in the liver at this time point using the above experimental protocol. Large (> 5 mm) nodules were physically separated from surrounding tissue and isolated cells were suspended in PBS and prepared for transplantation experiments. Prior to transplantation, both normal and nodular hepatocyte suspension was filtered through a nylon mesh with a pore diameter of 100 μ, in order to eliminate any large cell clumps. Cell viability, determined by trypan blue dye exclusion, was 85–90% for normal hepatocytes and 80–85% for the nodular cell preparation. AL- or CR-fed DPPIV- recipients were injected with 2 × 10^6^ normal hepatocytes or 1.7 × 10^6^ nodular hepatocytes freshly isolated from DPPIV^+^ syngeneic donors. Cells were transplanted into the liver via a mesenteric vein.

### Histology and immunohistochemistry

To follow the fate of transplanted cells, histochemical detection of DPP-IV positive clusters was performed on 5 μm frozen sections as previously described [13]. Formalin-fixed, paraffin-embedded sections were stained with hematoxylin and eosin (H&E) according to standard procedures. Immunohistochemical staining for GSTP was performed on paraffin embedded sections, following de-wax and antigen retrieval with sodium citrate buffer (pH 6, 0.01M). Slides were blocked for 30′, incubated with primary antibodies (Anti-GSTP, LifeSpan Biosciences, Seattle, WA; C179188) overnight at 4°C, and then incubated with AP-conjugated secondary antibodies. Detection of specific signal was accomplished using the avidin/biotin alkaline phosphatase system (Vectastain ABC kit; Vector Lab, Burlingame, CA). Double staining of DPPIV and GSTP was performed on frozen sections fixed in acetic alcohol/ethylic alcohol, first stained for DPPIV (as reported above), then blocked with goat serum, incubated with anti-GSTP overnight at 4°C and detected with the same protocol for paraffin sections.

### Cell imaging analysis

Three dimensional analysis of DPPIV^+^ cluster was performed on 10 consecutive serial sections by scanning slides with Hammamatsu NanoZoomer 2.0 rs. Acquired images were overlaid and analyzed using NDP 2.0 view software. GSTP positive clusters analysis was performed in at least 2 random sections from each sample by scanning slides with Pathscan Enabler IV scanner (Meyer Instruments, Houston, TX, USA). Cell number and cluster size was analyzed on acquired images using Image-Pro Premier Software (Media Cybernetics, Rockville, MD, USA).

### Staining for SA-beta-gal activity

Staining for SA-β-gal was performed according to published procedures [50]. Briefly, X-Gal stock solution was prepared by dissolving 20mg/ml X-Gal (Invitrogen, Carlsbed, CA) in dimetylformamide, immediately before staining. SA-β-Gal staining solution was prepared as follows. One mg/ml of X-Gal stock solution were dissolved in 40 mM citric acid in sodium phosphate, pH 6.0/5 mM potassium ferrocyanide/5 mM potassium ferricyanide/150 mM NaCl/2 mM MgCl2. Frozen sections of 10-μm thickness were fixed for 5′ in 4% formaldehyde/0.5% glutaraldehyde at 4°C, washed in PBS and incubated in fresh SA-β-Gal staining solution for 16h at 37°C. Sections were counterstained with Hematoxylin.

### Western blot

Western blot analysis was performed either on nuclear or cytoplasmic proteins extracted from liver samples by using a commercially available kit (CelLytic nuclear extraction kit, Sigma) according to manufacturer's protocol. Protein concentration in supernatants was measured at 560 nm using the BCA method [51]. Samples (80 μg and 30 μg for nuclear and cytoplasmic proteins, respectively) were prepared in Laemmli buffer, boiled at 95°C for 5′ then loaded into SDS-PAGE precast gels (Bio-Rad, Hercules, CA) and run under denaturing conditions. Proteins were transferred onto nitrocellulose membranes (GE Healthcare Life Sciences, Chicago, IL), blocked with 5% non-fat milk for 45′, followed by incubation with primary antibodies overnight at 4°C. Antibodies were as follows: p16 (sc1207), p21 (sc-471), p27 (sc-528), p53 (sc1311), SIRT1 (sc15404), HDAC1 (sc7872), C/EBPβ (sc150), (all from Santa Cruz, Santa Cruz, CA); 53BP1 (ab87097), Actin (ab8227) (all from Abcam, Cambridge, UK); FOXO1 (C29H4, Cell Signaling Technology, Danvers, MA). Membranes were washed and incubated for 2 h with the appropriate secondary antibody conjugated with HRP. Protein bands were detected using a chemiluminescent substrate (Bio-Rad) and imaged onto Kodak film.

### Graphical representation of results and statistical analysis

All results and statistical analysis were computed using Graph Pad Prism 5 (GraphPad Software, La Jolla, CA). Student “t” test analysis was performed to compare two groups of data. For cluster size distribution, statistical analysis of frequency distribution was performed via Chi-square test for contingency.
